# Mindfulness-Based Interventions for the Reduction of Postoperative Pain in Hip and Knee Arthroplasty Patients: A Systematic Review and Meta-Analysis

**DOI:** 10.7759/cureus.40102

**Published:** 2023-06-07

**Authors:** Michael F Barton, James Groves, Borna Guevel, Kirin Saint, Brenna L Barton, Mahmoud Hamza, Stefania I Papatheodorou

**Affiliations:** 1 Emergency Medicine, Harvard Medical School, Boston, USA; 2 Health and Social Behavior, Harvard School of Public Health, Boston, USA; 3 Quantitative Methods, Harvard School of Public Health, Boston, USA; 4 Internal Medicine, University of Michigan Medical School, Ann Arbor, USA; 5 Emergency Medicine, Tufts University School of Medicine, Boston, USA; 6 Epidemiology, Harvard School of Public Health, Boston, USA

**Keywords:** surgery, orthopedics, total joint arthroplasty, opioid, pain, postoperative pain, meditation, mindfulness

## Abstract

Purpose: The aim of this systematic review and meta-analysis is to evaluate the effect of mindfulness-based interventions (MBIs) on post-surgical pain in patients undergoing a total hip replacement (THR) or total knee replacement (TKR).

Methods: We performed a systematic review and meta-analysis in accordance with the Preferred Reporting Items for Systematic Reviews and Meta-Analysis (PRISMA) guidelines. A search of multiple databases, including PubMed and EMBASE, was performed for studies from database inception through March 2nd, 2022. Data were extracted, and pooled estimates of standardized mean differences in pain scores were calculated using a random effects model and inverse probability weighting.

Results: Two randomized control trials were eligible for inclusion (299 patients). The average ages of participants in each study were similar at 65.5 and 64.8 years, and both studies were predominantly female at 72.4% and 61.9%. The mindfulness intervention ranged from an eight-week program to a 20-minute session. Both individual studies reported statistically significant reductions in postoperative pain for MBI groups. The pooled standardized mean difference in pain scores for the MBI groups compared to the control groups was -1.94 (-3.39; -0.48).

Conclusions: There exists preliminary evidence for the beneficial effect of MBIs on reducing the postoperative pain experience in this patient population. Given the significant consequences of postoperative pain and the necessity for non-opioid forms of analgesia, this topic represents a promising area of research that warrants future randomized control trials to better understand the role of MBIs for postoperative analgesia.

## Introduction and background

Total knee replacement (TKR) and total hip replacement (THR) are common orthopedic procedures for end-stage osteoarthritis and rheumatoid arthritis and are performed more than 1 million times per annum globally [1]. While highly effective treatment options, these procedures result in moderate or severe postoperative pain for most patients [2]. If poorly controlled, this pain may worsen postoperative outcomes by preventing early ambulation and joint mobilization, increasing the risk of thromboembolism [3], and reducing patient well-being and satisfaction [2]. 

Current strategies to control postoperative pain after TKR and THR rely heavily on opioid medications. However, adverse effects are common within such analgesic regimes [4] and opioid use disorders are increasingly prevalent with the United States experiencing an escalating public health crisis termed the “Opioid Epidemic” [5] related to sharp rises in recent years in opioid-related hospitalizations [6] and opioid overdose death rates [7]. Chronic opioid usage is common after surgery, ranging from 4.4%-23.8% depending on the subspecialty, and is most common for those undergoing orthopedic procedures (23.8%) [8]. The development of non-pharmacological methods for postoperative analgesia in TKR and THR is crucial in reducing reliance on opioids in the postoperative phase and preventing the adverse consequences related to opioid use disorders, whilst also maintaining good surgical outcomes and patient satisfaction.

Mindfulness is defined as “the awareness which arises from purposefully paying attention to present moment experiences non-judgementally” [9]. Mindfulness-based interventions (MBIs) involve training in mindfulness meditation, during which participants learn to relate to negative experiences, such as painful stimuli, in a receptive, open, and non-judgmental manner [10]. It has been theorized that interfacing with pain in this manner may result in the attenuation of its severity. Evidence from neuroimaging studies has indicated that MBIs may reduce the discomfort of painful stimuli through changes in neurophysiological patterns within the cerebral cortex in response to thermal pain [11], which differ from those observed during placebo [12]. 

MBIs have been utilized in the clinical setting as an effective non-pharmacological means of reducing musculoskeletal pain related to rheumatoid arthritis [13], fibromyalgia (Cohen d effect size, 0.4-1.1) [14], and chronic low back pain (relative risk of clinical improvement 1.37 (1.06-1.77) for mindfulness-based stress reduction (MBSR) vs. usual care) [15]. Recent work has demonstrated that MBIs may show promise for reducing postoperative pain after septorhinoplasty [16]. 

Current studies on this subject include small sample sizes and some null results can be the result of a lack of statistical power. A meta-analysis can address these concerns and combine the results of individual studies to boost power. Further, a systematic review allows for the qualitative synthesis of the existing literature on this subject. The aim of this systematic review and meta-analysis is to evaluate, based on the existing literature, the effect of MBIs on post-surgical pain in patients undergoing a THR or TKR.

## Review

Methods

Protocol and Registration

We performed a systematic review and meta-analysis in accordance with the Preferred Reporting Items for Systematic Reviews and Meta-Analysis (PRISMA) Guidelines [17]. The protocol was registered with PROSPERO (registration number CRD42022316344).

Eligibility Criteria

Studies were considered eligible if they were randomized clinical trials or observational studies that investigated the impact of mindfulness-related interventions on post-surgical pain in patients undergoing a THR or TKR. Studies that employed interventions such as yoga, tai chi, qigong, and transcendental meditation or relaxation therapy without explicit reference to mindfulness were excluded. Studies that employed interventions that did not include formal mindfulness meditation practice, such as Acceptance and Commitment Therapy, were excluded. 

Databases

We built the search strategy, without language restriction, for PubMed, EMBASE, PsycINFO, Web of Science, Cochrane, and ClinicalTrials.gov for all articles published from database inception to March 2nd, 2022. 

Search Strategy

A comprehensive search strategy was created with the support of a librarian at the Harvard Countway Library of Medicine. Our search terms included terms for mindfulness, meditation, mind-body therapies, breathing exercises, relaxation therapy, cognitive behavioral therapy, and arthroplasty. Our full search strategy is included in our appendix.

Study Selection

Search results from all sources were uploaded to Covidence. Duplicate studies were removed. Two reviewers (M.B. and J.G.) independently screened titles and abstracts and then reviewed full texts. Inconsistencies at each step were resolved by discussion and reaching a consensus. The reference lists of included studies were also reviewed for relevant articles. 

Data Collection Process and Data Items

As per the pre-study protocol, data extraction was performed by one author (M.B.) and checked by another (B.B.). Elements that were extracted from each study included the following: study reference, study design, study demographics, intervention details (length and type of training), control details (usual care vs. other), pain outcome data (mean and standard error for group scores or group differences), and the number of patients in each group (control vs. intervention). It was determined ahead of time that correspondence with study researchers would be sought via email to request any unreported data or details, if necessary.

Risk of Bias in Individual Studies

For our study-level quality assessment, we a priori specified that we would use the Cochrane RoB 2 (risk of bias 2) tool and the ROBINS-I (“Risk Of Bias In Non-randomised Studies--of Interventions”) tool for randomized control trials and observational studies, respectively, to assess the quality of our studies and risk of bias [18,19]. Given that there were no observational studies in our review, the ROBINS-I tool was not used. Per the RoB 2 protocol, we assessed trials on random sequence generation, allocation concealment, blinding of participants and health care personnel, blinded outcome assessment, completeness of outcome data, evidence of selective reporting, and other biases. Risk of bias assessments were performed independently by two authors (M.B. and K.S.). Disagreements were resolved by discussion and reaching a consensus. A risk of bias plot was created using the Metafor package in R statistical software, version 4.1.2 (R Foundation for Statistical Computing, Vienna, Austria) [20,21].

Summary Measures

Standardized mean differences were used as the main effect measure for analyzing pain differences for intervention vs. control groups. A negative mean difference reflects that the mindfulness intervention resulted in a lower pain score compared to the control group. If a mean difference was not reported, we calculated it ourselves using the difference in mean pain scores for intervention and control groups. In order to calculate standard errors (SE) for our effect measures, we utilized reported 95% confidence intervals (CI) and reverse-calculated standard errors using the equation: SE = (CI upper bound - mean)/1.96 (assuming a normal distribution of pain scores) [22]. In anticipation of the use of different pain scores, we standardized our mean differences for pain by dividing our mean differences by their respective standard errors. 

Synthesis of Results

We created pooled estimates of our effect measures (standardized mean differences) using a random effects model utilizing inverse probability weighting [23]. Restricted maximum likelihood estimation was used to estimate the variance of the effect sizes in our random effects model. Heterogeneity was assessed by eyeballing the forest plots as well as I^2 statistics. If pain outcome data was present at more than one timepoint in a study, we a priori specified that we would utilize data from the timepoints that were shared/most similar across studies in order to maximize consistency.

Given the limited number of studies and data, we were unable to perform other types of analyses, including publication bias analysis, sensitivity analysis, subgroup analysis, or metaregression. Statistical analyses were performed using the Metafor package in R statistical software, version 4.1.2 [20,21]. All comparisons were two-tailed utilizing a threshold of p = 0.05 for significance.

Results

Study Selection

**Figure 1:**
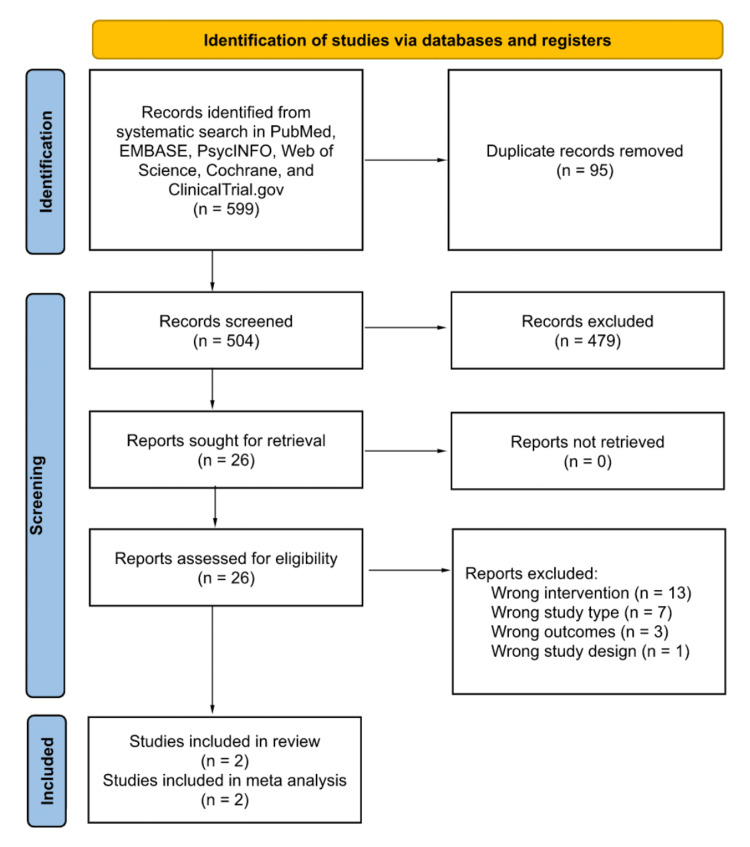
Preferred Reporting Items for Systematic Reviews and Meta-Analysis (PRISMA) Flow Diagram

Our systematic search of articles published before March 3rd, 2022 and related to our study question identified 504 unique results. After title and abstract screening, we were left with 26 studies that we considered potentially relevant. Two studies remained after full-text review (reasons for exclusion included “wrong intervention” (13 articles), “wrong study type” (seven articles), “wrong outcomes” (three articles), and “wrong study design” (one article)). Two studies reported mean differences in pain scores and were included in the meta-analysis. For our full PRISMA flow diagram, please see Figure 1.

Study Characteristics

Table 1 describes the included studies in terms of their study population, intervention, comparison groups, and outcomes.

**Table 1:** Study Characteristics

Study	Dowsey et al. 2019 [25]	Hanley et al. 2021 [24]
Study type	Single-site Randomized Control Trial	Single-site Randomized Control Trial
Medical center	St. Vincent’s Hospital, Melbourne, Australia	Department of Orthopaedic Surgery, University of Utah, Salt Lake City, Utah, United States
Surgery type included	Total hip or knee arthroplasty	Total hip or knee arthroplasty
N randomized	127	172
Intervention type	Mindfulness-Based Stress Reduction (MBSR) group-based program with a qualified MBSR facilitator and standardized curriculum	1. Mindfulness of breath instruction encouraging focused attention on breath and acceptance of thoughts, emotions, and sensations. 2. Mindfulness of pain instruction encouraging shifting attention from unpleasant to pleasant sensations.
Intervention timing	8-week-long pre-surgery program with weekly 2.5-hour sessions and a 7-hour full-day session. One ‘booster’ day-long workshop 3 months post-surgery	One 20-minute pre-surgery session
Control	Treatment as usual (TAU)	TAU + cognitive-behavioral pain psychoeducation intervention (one 20-minute pre-surgery session)
Primary outcomes of study	Post-operative pain and physical function	Pain intensity, pain interference, and opioid use
Pain measurement tool	Western Ontario and McMaster Universities (WOMAC) Osteoarthritis Index	Numeric rating scale from 0-10
Pain measurement timepoints	3 and 12 months	Postoperative days 2, 3, 7, 14, 21, and 28

Study population: Both studies were recently published open-label randomized control trials utilizing patients receiving either a total hip or knee arthroplasty. Hanley et al. 2021 recruited patients at the University of Utah’s Department of Orthopaedic Surgery in Salt Lake City, Utah, USA [24], while Dowsey et al. 2019 recruited patients from St. Vincent’s Hospital in Melbourne, Australia [25]. There were 299 patients total across both studies that were included in the randomization process and a combined total of 228 patients were retained at follow-up. For the trial arm sizes for each study, please see Table 2. With regards to the sociodemographics of the participants, the average ages of participants were similar at 65.5 and 64.8 years. Both study samples were predominantly women. Eligibility criteria varied between the studies. Dowsey et al. 2019 included THR/TKR patients aged 18 years or older who had English language proficiency and had a Short Form-12 survey mental component summary score <40 (in an effort to identify patients with pre-operative psychological distress who they argued would be at risk for sub-optimal symptom improvement following THR/TKR and would be best placed to benefit from MBIs). Those who lacked the capacity to consent were receiving surgery for neoplastic disease, had limited English proficiency, or had been diagnosed with a substance use disorder were also excluded. Hanley et al. 2021 had less selective eligibility criteria and included all individuals 18+ scheduled for THR/TKR who were English-speaking and did not experience significant surgical complications or consecutive surgeries. 

**Table 2:** Summary of Individual Study Results

	Dowsey et al. 2019 [25]	Hanley et al. 2021 [24]
Number Randomized	Total: 127	Total: 172
			Mindfulness of Breath: 41
				Mindfulness of Breath: 65
		Mindfulness of Pain: 76		
	Control: 62			
			Control: 55	
		Number Retained at Follow-up		Total: 110	Total: 118
						Mindfulness of Breath: 34
	Mindfulness of Breath: 44					
			Mindfulness of Pain: 45			
				Control: 56
					Control: 39
Mean Age (years)	65.5		64.8		
Percent Female	72.40%		61.90%		
Mean BMI	33.1		30.8
Mindfulness Intervention Effects on Postsurgical Pain	Mindfulness of Breath: Significant reduction of -10.3 (-19.0, -1.6) pain units (on a scale of 0-100) at 12 months postoperative		Mindfulness of Breath: No significant pain reduction at postoperative days 3, 7, 14, 21, 28
			Mindfulness of Pain: Significant reduction of -1.8 (-2.84, -0.76) pain units (on a scale of 0-10) at postoperative days 14, 21, and 28

Intervention: Preoperative MBIs varied considerably in their duration and content. Dowsey et al. 2019 utilized a preoperative MBSR program [26]-a well-validated, comprehensive eight-week program that included weekly 2.5-hour sessions and a seven-hour full-day session in the sixth week of the program. Participants were encouraged to practice for 45 minutes per day between weekly sessions. Additionally, patients were offered a ‘booster’ day-long workshop three months post-surgery. On the other hand, the MBI utilized by Hanley et al. was considerably briefer: a single 20-minute preoperative session that consisted of a five-minute summary of the biopsychosocial aspects of pain followed by a 15-minute scripted pain management mindfulness meditation session. Of those receiving an MBI in this study, 34 of 79 participants were randomized to the Mindfulness of Breath (MoB) condition where the meditation session guided participants in paying non-judgemental attention to sensations associated with breathing. The remaining 45 patients in the intervention group were randomized to the Mindfulness of Pain (MoP) condition where the meditation session guided participants to shift attention from unpleasant to pleasant sensations. Audio recordings were provided so patients could repeat the meditation sessions as necessary.

Control: Dowsey et al. 2019 utilized a ‘treatment as usual’ (TAU) comparison group, where participants underwent THR/TKR without any additional non-pharmacological methods of analgesia. Hanley et al. 2021, on the other hand, used an active comparison group in the form of a 20-minute preoperative cognitive-behavioral pain psychoeducation (CB) intervention which consisted of education about the link between thoughts, emotions, and behavior and provided instruction on how to modify maladaptive thoughts and behaviors regarding pain that might otherwise exacerbate pain and distress. When Hanley et al. were contacted about their reasoning for choosing a CB control group, they stated they originally had planned to include CB as well as TAU control groups; however, they removed the TAU group at the direction of reviewers at a top-tier general medical journal (ultimately rejected for unrelated reasons). All groups, including the MBI groups, received comparable baseline standard medical care upon which psychological interventions were added or not (group-dependent). Standard baseline analgesia included intraoperative local infiltration, regional nerve blocks, Tylenol, non-steroidal anti-inflammatory drugs (NSAIDs), and opioids.

Outcomes: The primary outcomes in Dowsey et al. 2019 were physical function and pain intensity-for which they utilized the knee pain component of the Western Ontario and McMaster Universities (WOMAC) Osteoarthritis Index to measure self-reported pain at three months and 12 months post-surgery. In Hanley et al. 2021, they measured pain intensity using a 10-point scale on postoperative days two, three, seven, 14, 21, and 28. The other primary outcomes in Hanley et al. 2021 were paint interference and opioid use. Dowsey et al. 2019 and Hanley et al. 2021 both reported mean differences in pain intensity between the MBI and control groups. This summary statistic was utilized as the effect size for both studies. For our full table of study characteristics, please see Table 1.

Risk of Bias in Individual Studies

**Figure 2:**
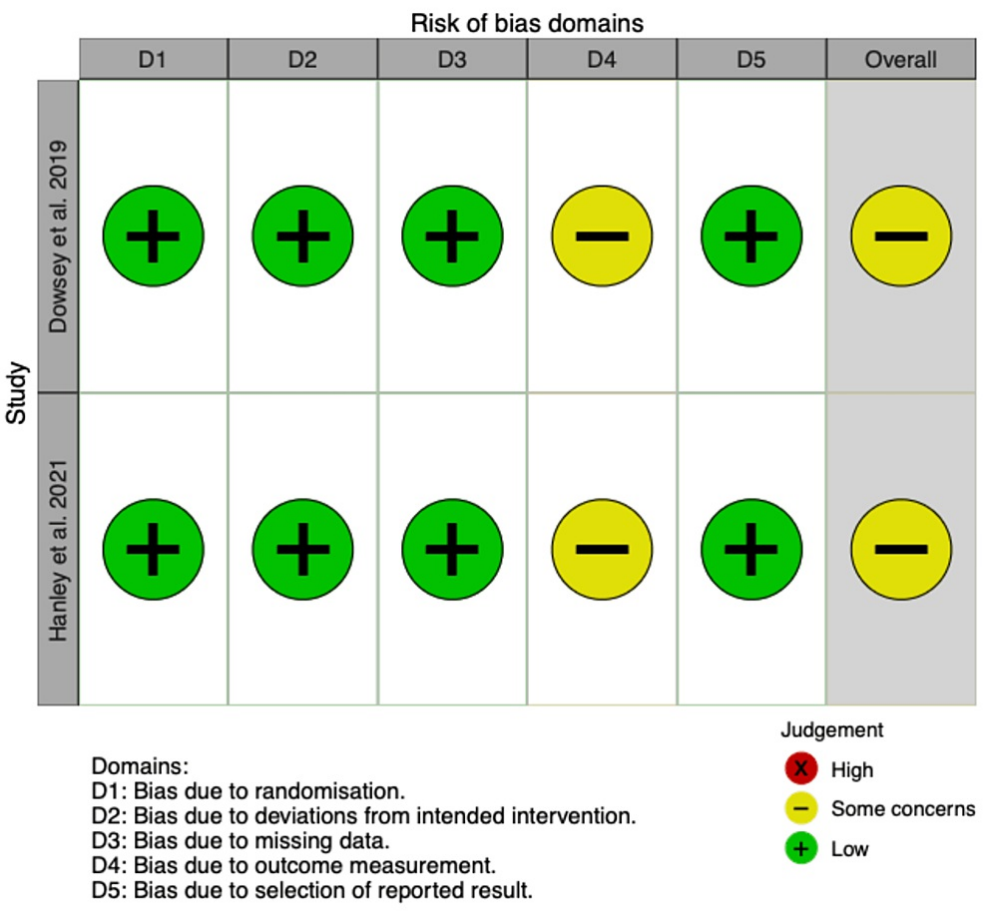
Risk of Bias Plot References: Dowsey et al. 2019 [25], Hanley et al. 2021 [24]

The risk of bias was assessed in both studies; please see Figure 2 for detailed RoB 2 scoring results for each study. The overall risk of bias was deemed “some concerns” in both studies. For both studies, this reflected a lack of treatment blinding-a condition that is challenging given the nature of the mindfulness interventions. Importantly, all other domains were deemed “low concern” for bias. Both studies were open-label randomized control trials. Both had a random allocation sequence that was concealed until patient assignment. Both studies included evidence that the results were not biased by missing data.

Mindfulness Interventions and Postoperative Pain: Individual Study Results

Table 2 summarises the individual study results of both of our included studies. Dowsey et al. 2019 observed that the eight-week MBSR program, delivered pre-operatively to THR/TKR patients, produced a statistically significant reduction in pain at 12 months but no reduction at three months, as compared to TAU. This reduction in pain at 12 months manifested as a mean difference of -10.3 (95% CI -19.0 to -1.6) on the WOMAC index pain subscale transformed to a scale of 0 to 100. Also observed was a statistically significant improvement in physical function on the 100-point WOMAC function score at 12 months post-surgery of 10.3 points (95% CI 1.3 to 19.2). Hanley et al. 2021 observed that the MoP intervention, delivered pre-operatively to a cohort of THR/TKR patients, produced a statistically significant reduction in pain intensity at postoperative days 14, 21, and 28 as compared to the CB group. On postoperative day 28, this manifested as a mean difference of -1.8 (95% CI -2.84 to -0.76) on a pain scale ranging from 0-10. No statistically significant difference in pain intensity ratings was seen between the MOB group compared to the CB group. The MOB group, however, did have a significant reduction in opioid use at postoperative day 21. 

Mindfulness Interventions and Postoperative Pain: Meta-Analysis

**Figure 3:**
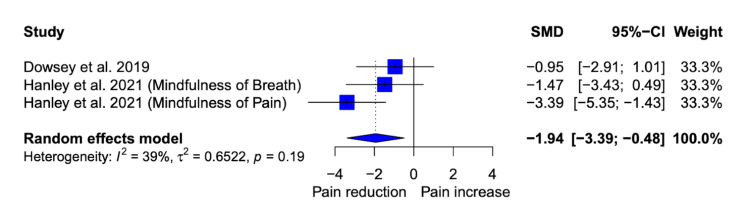
Mindfulness Interventions and the Reduction of Postoperative Pain: Forest Plot References: Dowsey et al. 2019 [25], Hanley et al. 2021 [24]

Both studies reported mean differences in pain scores for the MBI groups compared to the control groups. Of note, Hanley et al. 2021 consisted of two MBI groups (MoP and MoB), and we included both groups in our analysis. These two groups plus the MoB group in Dowsey et al. 2019 made for a total of three groups for our meta-analysis. We utilized the three-month pain measurement timepoint from Dowsey et al. 2019 and the 28th postoperative day timepoint for Hanley et al. 2021 given they were the time points that were most similar to each other between studies (a strategy that was specified a priori). Overall, MBIs (MoP or MoB) were significantly associated with a decrease in standardized pain scores of -1.94 (-3.39; -0.48) compared to control groups that received no MBI (see Figure 3). Heterogeneity was moderate (I^2 = 39%) but not significant (p = 0.40). Given the concern for dependent effects resulting from the fact that two of our meta-analysis groups were from the same study, we also created a correlated and hierarchical effects model (CHE) [27] to allow for robust variance estimation all using the Metafor and clubSandwich packages for R software [20,21,27]. The results of this model did not differ substantially from our original model suggesting little concern for dependent effects and affirming the veracity of our original model. Additionally, for the CHE we assumed a rho correlation coefficient of 0.6 but a sensitivity analysis of rho values ranging from 0 to 1 showed little difference in the model results. Given the limited number of studies, we were unable to perform other types of analyses, including publication bias analysis, sensitivity analysis, subgroup analysis, or metaregression.

Discussion

This systematic review and meta-analysis suggests that preoperative MBIs may produce reductions in postoperative pain for patients undergoing TKR/THR as compared to TAU or cognitive behavioral therapy pain education. This result is consistent with the broader literature which has found MBIs to have analgesic effects in reducing pain arising from various conditions, including chronic pain, primary headaches, cancer-related pain, rheumatoid arthritis, fibromyalgia, and other types of postoperative pain [13-16,28-31].

Secondarily, one study in our review, Dowsey et al. 2019, found that MBIs may also be associated with improvement in several other outcomes in addition to pain reduction including physical function. While there is still limited evidence for the associations between MBIs and postoperative physical functioning, the link between psychological distress and physical functioning after surgery is more established [32-34]. Through reducing psychological distress, MBIs may therefore also significantly affect postoperative functional outcomes scores. Other potential mechanisms of action for MBIs improving postoperative physical function would be an interesting area of future research. 

Need for Non-Opioid Analgesia for Orthopaedic Postoperative Pain

TKR and THR are among the most common procedures in the United States [1]. Unfortunately, postoperative pain associated with a total joint arthroplasty (TJA) is a significant burden for patients and is especially severe for those who underwent a TKR [1]. Over half of the patients undergoing these procedures report moderate to severe pain levels [2].

Postoperative pain has negative consequences that go beyond the patient’s pain experience itself. Pain after a TJA leads to a myriad of downstream effects that impede patient well-being. Postoperative pain can exacerbate and prolong the endocrine and neurologic “stress response” which can result in compromised immunity as well as cardiovascular strain and increased incidence of ischemic cardiac events [35]. Pain also leads to postoperative functional immobilization which can cause an increased risk of decreased pulmonary function, small bowel ileus, and thrombus formation [3,36]. In addition to physical health effects, patients with inadequately controlled pain can be at higher risk for mental health consequences, including anxiety and delirium [37]. All of this leads to an increased burden on providers and the healthcare system as a whole, in addition to the patient.

The mainstay of postoperative pain management has been opiate medications. However, opiate use is fraught with many postoperative complications and health risks, including progression to misuse, dependence, and overdose [5,38]. In addition, there is evidence that opiates alone are not associated with adequate postoperative pain control [39].

Clearly, alternative ways of addressing postoperative pain for TJA are needed. The strategy of preemptive (initiated before surgery) and multimodal (using two or more analgesic agents with differing mechanisms of action) analgesia has been recommended as a way to decrease complications while providing the best analgesia for patients [37]. MBIs represent a promising new analgesic strategy for TJA. Not only have MBIs shown effectiveness for meaningfully reducing pain in other conditions [13-16,28-31], but they are also considered to be safe and largely free of side effects and can be readily accessible at any time to patients trained in MBI techniques. Further, there is evidence that MBIs even reduce the risk of opioid cravings, misuse, and dependence [40], making them attractive options to support the safe use of opioid analgesia as well.

Mechanism of Action of MBIs for Analgesia

The experience of pain is complex, possessing both biological and psychological components. Pain is a protective somatosensory phenomenon first initiated by peripheral nociceptors at the site of tissue damage and then transmitted through the dorsal horn and spinothalamic pathway of the spinal cord into cerebral regions, which include the thalamus, somatosensory cortex, anterior cingulate cortex, and prefrontal cortex [11]. It is crucial to note that the severity of pain felt is not necessarily solely determined by the characteristics of the pain-inducing stimulus itself. Cognitive and emotional factors (such as experiences, mood, expectations, and desires) also play an important role in determining pain severity and modifying the transmission of pain through the aforementioned higher-level brain structures.

Research suggests that mindfulness practices can alter the cognitive processes involved in pain processing, which leads to changes in pain perception [11]. Specifically, MBIs encourage the user to react neutrally and nonjudgmentally to various experiences, including pain. This more non-judgemental cognitive schema can reduce the acute pain experienced by reducing reactivity to distressing feelings that may amplify the pain experience [11]. One concrete example of this draws from the “pain perception as inference” theoretical framework. This framework states that it is common for patients to use inferences from previous pain experiences to inform future experiences with pain and to create schemas, such as “My pain will never get better,” which can prolong and exacerbate their pain episodes [41]. Altering this schema with MBIs can help promote patient pain reduction.

These cognitive schema modifications may lead to biological changes in pain processing, with evidence indicating that mindfulness practices attenuate pain by altering pain processing at the orbitofrontal cortex, anterior insula, anterior cingulate cortex, and thalamus [11,12]. This alteration of brain-region-specific activation is also coupled with changes in various endogenous neurochemical modulatory systems, including opioidergic, cannabinoid, serotonergic, and dopaminergic systems [11].

Future Directions

Further evaluation of the relationship between MBIs and the magnitude and timing of their analgesic effect is needed. Whereas Dowsey et al. 2019 observed no significant pain reduction in the MBI group at three months, pain reduction was observed in the MoP group of Hanley et al. 2021 as early as postoperative day 14. Additionally, it would be helpful to better understand how the duration of MBIs might impact their level of analgesic effect. Dowsey et al. 2019 evaluated an eight-week intensive program while Hanley et al. 2021 utilized brief, 20-minute sessions undertaken three weeks before surgery. Both studies found significant pain reduction among their participants despite dramatically different program lengths and resource utilization. These results indicate that even brief interventions may be highly effective. In future years, it will be important for studies to establish whether a dose-response relationship between the duration of MBIs and postoperative pain reduction exists. Similarly, future research is needed to assess the analgesic effect of different mindfulness practices. Interestingly, Hanley et al. 2021 found the two mindfulness intervention groups studied produced different results as compared to the CB control. While the MoP practice was observed to produce a strong and consistent analgesic effect, the MoB practice failed to show any statistically significant reductions in pain versus the control group. Hanley et al. 2021 postulated that MoP intervention had more of an effect on cognitive factors that prolong postoperative pain, such as pain catastrophizing, thus allowing it to have the strongest analgesic effect postoperatively. Additionally, the two studies included in our review both reported combined data for patients undergoing THR or TKR. In future studies, reporting joint-specific subgroup analyses as well might also be useful to facilitate subsequent subgroup analyses at the meta-analysis level. Finally, further investigation is warranted to explore MBIs role in promoting other postoperative health and recovery outcomes. Dowsey et al. 2019, along with other studies, provide evidence that MBIs delivered preoperatively to TJA patients may result in enhanced postoperative physical function [42]. Further, MBIs have been shown to improve psychiatric conditions [43] which are highly prevalent in surgical candidates and are associated with poorer surgical outcomes [44].

Strengths and Limitations

A limitation of our study is the small number of published studies that had been conducted addressing our specific question. Given this, no definitive conclusions regarding the analgesic effect of MBIs for TKR/THR patients can be made currently. However, the results of the individual studies show the analgesic potential of MBIs for this specific application. Both studies were high-quality randomized control trials, and both demonstrated a statistically significant and clinically-relevant reduction in postoperative pain resulting from MBIs, as compared to the control condition, for THR/TKR patients. Furthermore, this study employed a detailed pre-study protocol, thorough search strategy, robust risk of bias assessment, and advanced meta-analysis techniques that included robust variance estimation. 

Another weakness of our study was the heterogeneity of our intervention and control groups across our individual studies. While it is true that the supervised mindfulness interventions across each study differed in terms of length (one session vs. eight weeks), additional self-directed practice of the mindfulness techniques after the teaching session may have made the actual intervention durations more similar than it would initially appear. Further, we believe that the interventions were similar in terms of content. Specifically, while there can be many definitions of mindfulness, both studies defined mindfulness in a similar way with an emphasis on awareness and acceptance. Additionally, while our control groups were also somewhat different (treatment as usual vs. cognitive behavioral interventions), we felt they were similar to each other as well as both very different from the mindfulness interventions. Given all of this, we felt that a meta-analysis was appropriate, especially with the mixed effects model which allows us to account for the possible heterogeneity of effect sizes.

## Conclusions

While there is evidence of the benefits of MBIs for analgesia in various health conditions, this systematic review is the first of its kind to analyze the effectiveness of MBIs for the reduction of postoperative pain in THR/TKR patients. Our study suggests there may be a beneficial effect of MBIs on reducing postoperative pain in this patient population. Given the significant consequences of postoperative pain and the necessity for non-opioid forms of analgesia, this topic represents a promising new area of research that warrants future randomized control trials to better understand the role of MBIs for postoperative analgesia.

## Appendices

Search strategies for each database

**Table 3:** Search Strategies for each Database

Database	Search Strategy
*PubMed*	("Arthroplasty, Replacement"[Mesh:NoExp] OR "Arthroplasty, Replacement, Knee"[Mesh] OR "Arthroplasty, Replacement, Hip"[Mesh] OR hip replacement*[tiab] OR knee replacement[tiab]* OR joing replacement[tiab] OR hip arthroplast*[tiab] OR knee arthroplast*[tiab] OR joint arthroplast*[tiab] OR joint surg*[tiab] OR hip surg*[tiab] OR knee surg*[tiab]) AND ("Mindfulness"[Mesh] OR "Meditation"[Mesh] OR “Mind-Body Therapies”[Mesh:NoExp] OR "Breathing Exercises"[Mesh] OR "Relaxation Therapy"[Mesh] OR “Spiritual Therapies”[Mesh:NoExp] OR “Mental Healing”[Mesh] OR "Cognitive Behavioral Therapy"[Mesh] OR “Behavior Therapy”[Mesh:NoExp] OR mindful*[tiab] OR mbsr[tiab] OR mbct[tiab] OR mindfulness-based*[tiab] OR meditat*[tiab] OR mind body*[tiab] OR mind-body*[tiab] OR breathing*[tiab] OR relaxation[tiab] OR spiritual therap*[tiab] OR cognitive behavior*[tiab] OR cognitive behaviour*[tiab] OR cognitive therap*[tiab] OR behavior therap*[tiab] OR behaviour therap*[tiab] OR cbt[tiab])
*Embase*	('hip arthroplasty'/exp OR 'knee arthroplasty'/exp OR 'arthroplasty'/de OR ((hip OR hips* OR knee* OR joint) NEAR/3 (replacement* OR arthroplast* OR surg*)):ab,kw,ti) AND ('mindfulness'/exp OR 'meditation'/exp OR 'cognitive therapy'/exp OR ‘behavior therapy’/exp OR ‘relaxation training'/exp OR ‘breathing exercise’/exp OR ((cognitive OR behaviour* OR behavior*) NEAR/3 (therap* OR train*)):ab,kw,ti OR ((relax* OR breathing) NEAR/3 (train* OR exercise OR therapy OR intervention OR technique)) OR (mindful* OR meditat* OR cbt OR mbsr OR mbct OR ‘mind body’ OR mind-body):ab,kw,ti)
*PsycINFO*	DE "Surgery" AND (DE "Mindfulness" OR DE "Mindfulness-Based Interventions" OR DE "Meditation" OR TI mbsr OR AB mbsr OR TI mbct OR AB mbct OR TI mindful* OR AB mindful* OR TI meditat* OR AB meditat*)
*Web of Science*	(TS=(mbsr) OR TS=(mbct) OR TS=(mindfulness) OR TS=(meditation)) AND (TS=(hip arthroplasty) OR TS=(knee arthroplasty))
*Cochrane*	(mindfulness OR meditation OR mbsr OR mbct):ti,ab,kw AND (arthroplasty):ti,ab,kw
*Clinical Trials*	Arthroplasty AND (mindfulness OR meditation OR mbsr OR mbct)
